# Dynamic Association between HIV-1 Gag and Membrane Domains

**DOI:** 10.1155/2012/979765

**Published:** 2012-07-05

**Authors:** Ian B. Hogue, G. Nicholas Llewellyn, Akira Ono

**Affiliations:** ^1^Department of Microbiology and Immunology, University of Michigan Medical School, Ann Arbor, MI 48109, USA; ^2^Department of Molecular Biology, Princeton University, Princeton, NJ 08544, USA; ^3^Cellular and Molecular Biology Program, University of Michigan Medical School, Ann Arbor, MI 48109, USA; ^4^Department of Molecular Microbiology and Immunology, University of Southern California, Los Angeles, CA 90033, USA

## Abstract

HIV-1 particle assembly is driven by the structural protein Gag. Gag binds to and multimerizes on the inner leaflet of the plasma membrane, eventually resulting in formation of spherical particles. During virus spread among T cells, Gag accumulates to the plasma membrane domain that, together with target cell membrane, forms a cell junction known as the virological synapse. While Gag association with plasma membrane microdomains has been implicated in virus assembly and cell-to-cell transmission, recent studies suggest that, rather than merely accumulating to pre-existing microdomains, Gag plays an active role in reorganizing the microdomains via its multimerization activity. In this paper, we will discuss this emerging view of Gag microdomain interactions. Relationships between Gag multimerization and microdomain association will be further discussed in the context of Gag localization to T-cell uropods and virological synapses.

## 1. Introduction

Microdomain-based compartmentalization of the plasma membrane is implicated in many aspects of the HIV-1 life cycle. In particular, during events in the late phase of the HIV-1 life cycle such as assembly and cell-to-cell transmission, these microdomains have been thought to serve as preformed platforms that facilitate concentration of viral components (e.g., Gag and Env) or delivery of these proteins to specific locations in cells. However, recent studies suggest that Gag is not a simple passenger of microdomains but rather plays an active role in reorganizing microdomains via its membrane-binding and multimerization activities. In this paper, we focus on recent findings on this active role played by Gag during microdomain association. In light of this new view, we will also discuss the implications of plasma membrane microdomains and large-scale domains in cell-to-cell transmission. Microdomains are also thought to affect virion infectivity, attachment of virions to target cells, and virus-cell fusion, in which they modulate distributions and/or activities of Env, Nef, and virus receptors. For these topics, interested readers are referred to more comprehensive papers published in recent years [1–5].

## 2. HIV-1 Assembly at the Plasma Membrane

The viral structural polyprotein Gag is necessary and sufficient for the assembly of virus-like particles. HIV-1 Gag is synthesized as a 55 kDa polyprotein composed of 4 major structural domains (and 2 spacer polypeptides), as defined by cleavage by the viral protease: matrix (MA), capsid (CA), nucleocapsid (NC), and p6. However, proteolytic cleavage occurs largely after virion assembly and release; thus, its constituents must work together in the context of the full-length Gag polyprotein to drive particle assembly. After its synthesis in the cytosol, Gag traffics to the site of assembly, binds cellular membranes, multimerizes, buds through the membrane, and recruits host factors that mediate membrane scission, releasing an immature particle [6, 7]. It is increasingly apparent that many of these steps occur in a coordinated, interdependent fashion. Among them, Gag membrane binding and multimerization are implicated in association of virus assembly with membrane microdomains.

Gag membrane binding is mediated by its N-terminal MA domain, containing bipartite membrane binding motifs. The MA domain is cotranslationally myristoylated and contains a highly basic region (HBR) that binds the plasma-membrane-specific acidic phospholipid phosphatidylinositol-(4,5)-bisphosphate [PI(4,5)P_2_] [8–16] (reviewed in [17]). It has been suggested that exposure of the myristoyl moiety is regulated through a mechanism known as the myristoyl switch [18, 19]. Indeed, NMR studies demonstrated that prior to membrane binding, the myristoyl moiety is sequestered in a hydrophobic cavity of the MA domain. Upon Gag multimerization or PI(4,5)P_2_ binding, the myristoyl chain is exposed to promote membrane binding [14, 20, 21]. As for MA HBR, RNA appears to competitively regulate its binding to acidic membrane lipids. Studies using *in vitro* assays collectively support a model in which RNA bound to HBR prevents MA from binding to prevalent acidic lipids like phosphatidylserine, but allows MA binding to PI(4,5)P_2_, thereby enhancing the specificity of Gag binding to PI(4,5)P_2_-containing membranes, that is, the plasma membrane [10, 22–25]. 

Two major functional regions that contribute to Gag multimerization are the C-terminal region of the CA domain (CA-CTD) and NC. CA-CTD forms an interface that mediates Gag homodimerization [26–29]. The NC domain is thought to contribute to Gag multimerization via its ability to bind RNA [30–34]. Notably, heterologous leucine zipper dimerization motifs can substitute for NC in Gag multimerization and particle assembly [35–39]. These findings suggest a model in which RNA binding to NC serves a structural role, either as a scaffold or a trigger for CA dimerization. In addition to CA and NC, the Spacer Peptide 1 (SP1) between CA and NC plays an important role in regulating the multimerization process [40].

Higher-order Gag multimerization induces outward curvature of the plasma membrane area where the Gag multimer is bound. This step is likely driven by the inherent curvature of the Gag hexameric lattice, formation of which relies on CA [41]. Consistent with this, a number of CA mutations lead to a budding arrest phenotype, characterized by many electron-dense patches underneath the plasma membrane [29, 42]. Release of nascent particles is driven by the cellular ESCRT (endosomal sorting complexes required for transport) that is recruited to assembling virions through interactions with the NC and p6 domains [43].

HIV-1 has been observed to assemble at the plasma membrane in T cells and some laboratory cell lines such as HeLa cells (see [44] for a review). Assembly in macrophages was originally thought to occur at late endosomes/multivesicular bodies (LE/MVB), based on the apparently intracellular location of assembling Gag and the presence of LE/MVB markers, such as the tetraspanin protein CD63 and ESCRT [45, 46]. However, the sites of assembly in macrophages were found to be actually deep invaginations of the plasma membrane, now known as virus-containing compartments (VCC) (although the architecture of the VCC, in particular whether VCCs are all connected to the plasma membrane, is still under intense investigation) [47–53]. Moreover, markers like CD63 strongly colocalize with Gag, even at assembly sites that are unambiguously on the plasma membrane (e.g., [54, 55]). Therefore, the currently accepted idea is that for most cell types including macrophages, the primary site of HIV-1 assembly is the plasma membrane or its specific domains [56, 57].

## 3. Plasma Membrane Microdomains Associated with HIV-1 Assembly

The plasma membrane consists of diverse microdomains. This partitioning of membrane components is regulated by lipid-lipid, protein-protein, and protein-lipid interactions and compartmentalizes cellular processes [58]. As with many diverse enveloped viruses, HIV-1 was initially proposed to assemble at lipid rafts, based on sensitivity to cellular cholesterol depletion and cofractionation of viral components with detergent-resistant membranes (DRM). Subsequently, HIV-1 assembly was also proposed to occur at tetraspanin-enriched microdomains based on microscopy.

### 3.1. Lipid Rafts

Spontaneous partitioning of lipids into an ordered phase and a disordered phase has been observed in chemically defined model membranes and model membranes reconstituted from cellular membrane components [58]. The ordered phase is enriched in cholesterol and saturated lipids, and the disordered phase is enriched in unsaturated lipids. This biophysical phenomenon of lipid phase separation in model membranes has been hypothesized to underlie the phenomenon of lipid rafts in cells. In contrast to model membranes, however, cellular membranes contain a greater diversity of lipids and proteins. The partitioning of these molecules is governed by a much greater complexity of lipid-lipid, protein-lipid, and protein-protein interactions. Thus, the current consensus is that lipid rafts are highly dynamic, submicroscopic membrane domains enriched in sterols and sphingolipids, which can be stabilized to form larger platforms through protein-protein and protein-lipid interactions [58]. 

To assess the involvement of lipid rafts in HIV-1 assembly processes, biochemical assays that measure either resistance to nonionic detergents or sensitivity to cellular cholesterol depletion have been widely used. Results from these assays generally support lipid raft association of the HIV-1 assembly process [59–71]. Both cholesterol depletion and substitution of the Gag myristoyl moiety with an unsaturated acyl analogue inhibit virus particle production, suggesting a functional role for association between HIV-1 Gag and lipid rafts during virus assembly [66, 68, 69]. While biochemical methods used in these studies require cautious interpretations of data due to their inherent limitations [72–76], studies using different approaches, such as microscopy and virion content analyses described below, also generally support raft association with the HIV-1 assembly process.

Because of the dynamic and submicroscopic nature of lipid rafts, cross-linking of cell-surface proteins, which stabilizes the microdomains they associate with, is often used to observe protein partitioning into microdomains by standard fluorescence microscopy. When two microdomain markers are independently clustered using specific antibodies or toxins, these markers can colocalize within the same patch, or “copatch”, indicating propensity of these markers to partition into the same microdomains [77–80]. Consistent with biochemical analysis described above, Gag puncta that represent assembled particles or multimerizing Gag are observed to colocalize or copatch with raft markers, such as the glycosphingolipid GM1 and GPI-anchored proteins [42, 64, 67, 81–83]. However, a recent super-resolution microscopy study showed that GM1 does not colocalize with Gag clusters, at least in the particular cell type used [84]. Therefore, while GM1 may have a propensity to associate with lipid rafts, codistribution of this lipid with other raft components may occur only when raft partitioning is stabilized by crosslinking. These new super-resolution microscopy technologies will likely allow us to define the native distribution of each raft component associated with HIV-1 assembly sites. 

Finally, analyses of cellular molecules incorporated into HIV-1 particles also support lipid raft involvement during the HIV-1 assembly process. Biochemical, proteomics, and lipidomics studies have shown that the HIV-1 envelope is enriched in many of lipids and proteins that are also components of lipid rafts [85–91]. Of note, the cholesterol content of virions may be upregulated via activities of viral proteins such as HIV-1 Nef [92–94] and MLV glyco-Gag [95]. Importantly, by measuring spectral shift of the lipophilic fluorescent dye laurdan, which is sensitive to ordered packing of its surrounding lipids [96], HIV-1 envelope was shown to contain liquid-ordered domains [97].

### 3.2. Tetraspanin-Enriched Microdomains

Tetraspanin-enriched microdomains (TEMs) are plasma membrane microdomains organized by the homo- and heterooligomerization of tetraspanins, a family of homologous proteins with four transmembrane domains. Proteomics studies have identified a wide variety of proteins associated with TEMs. Most notably, tetraspanins interact with cell-adhesion molecules, integrins, and cell-signaling proteins, suggesting that TEMs serve as a platform to spatially organize cell-cell and cell-extracellular matrix adhesion and signaling [4, 98, 99]. Tetraspanins CD63 and CD81 have been shown to associate with phosphatidylinositol 4-kinase, a critical enzyme in creating a precursor for PI(4,5)P_2_ [100]. Importantly, different tetraspanins appear to be at least partially redundant in the cell functions measured in some of these studies.

The first evidence of association between tetraspanin proteins and HIV-1 assembly were early studies that found the tetraspanin protein CD63 enriched in the envelopes of HIV-1 particles. This was taken as evidence that Gag traffics through, or assembles at, an endosomal compartment, such as the LE/MVB. However, it was later shown that Gag associates with CD63 and other tetraspanin proteins at discrete microdomains on the plasma membrane [54, 55].

Tetraspanins, including CD9, CD63, and CD81, are incorporated into virus particles [45, 55, 88, 101–107], coimmunoprecipitate with Gag-laden cellular membranes [108], and strongly colocalize/copatch with Gag by immunofluorescence microscopy assays (e.g., [54, 55, 108]). As for functions, a variety of studies have suggested roles for tetraspanins and TEMs in different phases of the HIV-1 replication cycle such as virus entry (see [4] for a review). However, the role of tetraspanins and TEMs in Gag assembly remains currently unclear. The gross effects of perturbing tetraspanins by siRNA knockdown or overexpression are so far contradictory: some studies report perturbation reduces particle production [108, 109], while others report no effect [107, 110, 111]. In contrast, it is well accepted that tetraspanins incorporated into virus particles have an inhibitory effect on subsequent virus entry [107, 108, 110]. 

### 3.3. Gag Determinants for Interactions with Microdomains

While association of Gag with microdomains has been well documented, how this association occurs is only beginning to be elucidated. As saturated acyl chains mediate raft association of many cytoplasmic proteins, it is straightforward to imagine that the N-terminal myristoyl moiety of Gag plays a role. Consistent with this notion, incorporation of an unsaturated myristate analogue in the place of myristate impairs Gag recovery into DRM fractions [66]. Interestingly, an NMR study of MA bound to a soluble PI(4,5)P_2_ (with short acyl chains, allowing it to remain in aqueous solution) showed that, while PI(4,5)P_2_ binding induces myristoyl exposure, a hydrophobic cleft of the MA domain sequesters the typically unsaturated sn2 acyl chain of PI(4,5)P_2_—effectively exchanging an unsaturated acyl chain from PI(4,5)P_2_ for the saturated myristoyl chain of Gag [14]. This sequestration of the unsaturated sn2 acyl chain of PI(4,5)P_2_ has been hypothesized to facilitate Gag association with lipid rafts [14]. It remains to be seen if this acyl chain exchange occurs in the more authentic case of Gag binding a lipid bilayer, as opposed to interaction between isolated MA domains and water-soluble lipids. 

HIV-1 Gag multimerization has also been observed to enhance microdomain association. Biochemical studies showed that the presence of NC and other Gag regions necessary for multimerization affect the steady-state association of Gag with DRM [61, 65]. The presence of NC is also required for colocalization of Gag with markers for microdomains termed endosome-like domains (ELD), which appear to be a subset of TEMs [54, 112]. ELD association of Gag and other multimeric proteins was reported to be independent of a membrane-binding interface; a variety of plasma membrane targeting motifs were observed to mediate ELD association of a normally-cytosolic oligomeric protein, TyA [113]. Altogether, these results are consistent with a notion that Gag multimerization plays a key role in stable association with specific microdomains at the plasma membrane. 

In the context of assembly of many enveloped viruses, membrane microdomains are often regarded as preexisting platforms that accumulate viral structural components, thereby facilitating virus assembly. However, as alluded to earlier, protein-protein interactions are thought to stabilize or recruit microdomains [114, 115]. Therefore, Gag multimerization was postulated to modulate structure and/or size of Gag-associated microdomains [11, 65, 68, 86]. Consistent with this protein-centric view of microdomains, recent studies suggest that HIV-1 Gag is not just passively accumulated in microdomains but rather actively stabilize, recruit, or reorganize microdomains at the plasma membrane through its multimerization. Fluorescence recovery after photobleaching and single-molecule tracking analyses showed that Gag multimers trap the tetraspanin CD9 and, to a lesser extent, the raft markers GM1 and CD55 and clusters these microdomain components in a Gag-multimerization-dependent manner [82]. Furthermore, copatching and fluorescence resonance energy transfer analyses showed that HIV-1 coalesces TEMs and lipid rafts [42], two microdomains that are otherwise distinct and do not colocalize in cells that do not express Gag [98, 116–120]. Interestingly, correlative fluorescence and scanning electron microscopy showed that copatching between raft and TEM markers does not occur at assembly sites of a Gag mutant that forms multimeric Gag patches but fails to form spherical particles [42]. Therefore, raft-TEM coalescence appears to depend on membrane curvature induced by Gag multimerization. Altogether, Gag is likely to direct the formation of its own microdomains by recruiting and coalescing membrane proteins and microdomains, in a manner dependent on the process of virus assembly.

What determines microdomain recruitment to Gag multimers? Since MA functions as the interface of Gag with lipid bilayer, it is conceivable that MA or MA-interacting molecules drive recruitment of lipid raft and TEM markers. For example, the combination of the N-terminal myristoyl moiety and a saturated acyl chain of PI(4,5)P_2_, which is postulated to direct Gag to lipid rafts [14], may also direct small lipid rafts to Gag assembly sites. This is also consistent with the enrichment of specific lipids to the viral envelope, relative to the plasma membrane [86, 87]. However, copatching studies suggest that coalescence of lipid rafts and TEMs at assembly sites occur even when MA was replaced with a triple acylation motif or a heterologous lipid-binding domain [42]. Therefore, the MA sequence per se is not essential for reorganization of lipid rafts and TEMs. 

As described below, Gag multimerization is also important for Gag localization to larger membrane domains.

## 4. Large-Scale Membrane Domains Implicated in HIV-1 Spread

In addition to the microdomains described above, larger plasma membrane domains are implicated in HIV-1 spread. One of such domains is the VCC [47–53], which may serve as a virus reservoir that can transfer viruses upon contact with T cells [121–123]. A similar surface-accessible intracellular compartment in dendritic cells also promotes transmission of captured viruses to T cells via cell contacts during trans-infection [124–128]. In this section, however, we focus on membrane domains implicated in T-cell-to-T-cell virus transmission and their relationships with microdomains. 

### 4.1. Virological Synapses

HIV-1 virions released from infected cells may travel in the extracellular space until they come in contact with a target cell by chance (random three-dimensional diffusion and fluid flow). However, this cell-free infection route is much less efficient than cell-to-cell transmission, in which an infected cell physically contacts a target cell and directly transfers the virus. In contrast to cell-free transmission, cell-to-cell transmission of HIV-1 is 10- to several-thousand fold more efficient in cultured T cells [129–132] and is believed to be the major form of transmission for HIV-1 *in vivo*. In addition to HIV-1, direct cell-to-cell transfer is likely to be important for efficient spreading of several other retroviruses such as human T-lymphotropic virus-1 (HTLV-1) [133–136] and murine leukemia virus [137–139] as well as other pathogens (reviewed in [140, 141]). Moreover, a recent study suggested that cell-to-cell transmission enhances resistance of HIV-1 to antiretroviral drugs and therefore potentially constitutes a mechanism by which HIV-1 maintains an active reservoir in infected individuals undergoing combination drug therapy [142]. 

Cell-to-cell transmission occurs through several distinct plasma membrane structures. These structures include cytonemes [137–139], membrane nanotubes [143], and virological synapses (VSs) [124, 127, 134, 144–146]. Because involvement of membrane microdomains in the first two structures has yet to be described, in this paper we focus on VSs. VSs formed between HIV-1 infected and uninfected T cells are contact structures enriched in Gag, Env, and viral receptors. Stable VS formation between two T cells is primarily mediated by the Env-CD4 interaction [129, 145–148] unlike VS formed by monocyte-derived macrophages [121]. Consistent with this, antibodies that block the Env-CD4 interaction blocks VS formation and cell-to-cell virus transfer [129, 130, 145, 147, 149] (although neutralization by patient-derived antibodies is ineffective perhaps due to the delayed virion maturation during transfer at the VS [129, 144, 150]). The VS is also enriched in adhesion molecules such as LFA-1, although the significance of these adhesion molecules in VS formation and virus transfer/transmission varies depending on the experimental systems [146–148, 151]. 

VSs were first described for HTLV-1 [134]. Early studies including this HTLV-1 study and subsequent studies on HIV-1 VS have pointed to the importance of cytoskeleton in VS stability and formation [134, 145, 152–154]. Recent studies further suggest that polarization of HIV-1 Env is dependent on the microtubules and microtubule-dependent trafficking of secretory lysosomes that bear Env [155]. Consistent with this finding, Zap70, which regulates cell polarization in the immunological synapses by controlling localization of the microtubule organizing center (MTOC) [156], facilitates formation of VSs and cell-to-cell transmission [157]. The actin cytoskeleton is also important for VS formation, as evident from the impact of actin depolymerizing agents and a myosin light chain kinase inhibitor on VS formation, cell-to-cell virus transfer and transmission [145, 152, 158]. 

In addition to cytoskeleton, lipid rafts and TEMs are implicated in VS formation as well. Markers for both microdomains accumulate to VS [110, 146, 159, 160]. Consistent with a role for lipid rafts in VS formation, cholesterol depletion was observed to diminish formation of VS, as defined by the accumulation of CD4 (on the target cell) and HIV antigens (in the donor cell) at the cell-cell interface [159]. However, whether this impact was due to disruption of lipid rafts or inhibition of other cholesterol-dependent processes is unknown. If the former is the case, what particular role lipid rafts play in VS formation also remains to be determined. 

As for the role of TEMs, multiple and potentially opposing roles played by tetraspanins (for a review, see Thali [4]) make it difficult to assess the contribution of TEMs to VS formation. Anti-tetraspanin antibodies were observed to reduce VS formation albeit modestly [160]. Consistent with the inhibitory effect of tetraspanins on infectivity of virions [107, 108, 110], tetraspanins also prevent Env-mediated cell-cell fusion [161], inhibition of which was suggested to help preserve productive VSs [110]. Moreover, the presence of CD81, but not other tetraspanins, was shown to facilitate polarized localization of Gag [108]. On the other hand, CD81 was observed to decrease cell-to-cell virus transmission, perhaps via inhibition of virion infectivity [110]. Therefore, it remains to be determined whether and in what context TEMs or tetraspanins play a positive or negative role in cell-to-cell transmission of HIV-1 via VSs.

### 4.2. Uropods

**Figure 1:**
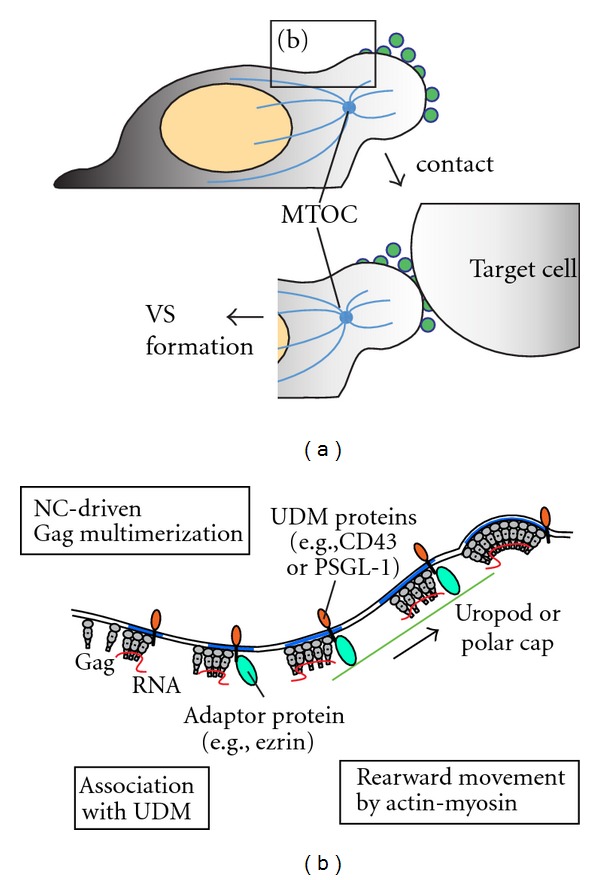
(a) Gag accumulates at the uropod surface. While it remains to be determined whether the first contact between virus-producing and target cells occurs right at the uropod or elsewhere during VS formation, virus-laden uropods do participate in VS formation as determined by concentration of uropod markers at the VS. (b) A working model for a mechanism by which Gag multimers associate with rearward actin flow that directs Gag to the uropod. NC-dependent Gag multimerization underneath the plasma membrane promotes association between Gag multimer and UDM. Of note, in contrast to lipid raft and TEM markers, UDM proteins appear to accumulate at assembly sites of wild-type Gag as well as those of a Gag mutant that multimerizes but fails to bud (GNL, unpublished data).

A majority of T cells in lymph nodes where cell-to-cell transmission likely occurs frequently are highly motile and adopt a polarized morphology [162–166]. The front end of a polarized T cell is called the leading edge, and the protrusion at the rear is called a uropod [167–169]. Functionally, uropods seem to promote T-cell migration by facilitating deadhesion of integrins such as LFA-1 that mediates substrate adhesion at the leading edge [169]. During T-cell migration, uropods also mediate contact with other T cells [170] and recruit bystander T cells to sites of inflammation [171]. Interestingly, Gag accumulates to the plasma membrane area constituting the uropod surface in polarized T cells [67, 129, 158] (Figure 1(a)). Moreover, upon contact with uninfected T cells, this plasma membrane domain participates in the VS, as supported by the observation that Gag and uropod markers on the infected cell and CD4 on the uninfected cell accumulate at the site of cell-cell contact [158]. These findings suggest a model in which the uropod surface of polarized HIV-1-infected T cells serves as a preformed platform that participates in VS formation.

 Which part of an HIV-1-infected cell mediates the initial contact with a target cell remains to be determined. It is possible that uropods, where the virus is concentrated, establish the initial contacts, and these contacts eventually develop into VSs without large-scale shift in cell polarity (as shown in Figure 1(a)). Consistent with this possibility, uropods are enriched in various adhesion molecules that help promote contact with other cells. However, it is also possible that initial contacts may be established at other regions of cells such as the leading edge. Under this scenario, after initial contact, viral proteins and VS components that are preaccumulated at the uropod would subsequently move laterally to the cell-cell junction. In support of this latter possibility, patches containing HIV-1 Gag have been observed to move laterally over the cell surface to the VS [144, 146]. Regardless of the pathways taken by Gag to the uropod and cell junctions, this preaccumulation of Gag at the uropod may constitute an important early step in VS formation.

The molecular mechanisms of Gag localization to the uropod also remain to be determined. Notably, Gag accumulation to uropods requires higher-order multimerization driven by NC [158, 172], while the dimerization function of CA-CTD is neither sufficient nor necessary [158]. In this regard, it is important to note that crosslinking of cell surface proteins with antibodies induces polarized localization of these proteins in leukocytes and other cell types [173]. Such “capping” has also been observed for lipids cross-linked by pentavalent cholera toxin [174, 175]. During T-cell polarization, similar polar cap formation occurs spontaneously at the cell surface from which a uropod originates [169, 176]. These capping phenomena depend on myosin II-driven rearward actin flow [177–181]. Thus, in a manner similar to capping, higher-order Gag multimerization might trigger Gag association with the actin flow, which in turn drives accumulation of Gag in uropods. In support of this model (Figure 1(b)), a myosin light chain kinase inhibitor ML7, which inhibits myosin II, dispersed Gag all over the cell surface [145, 158]. 

The nature of the link between Gag multimers and retrograde actin flow is currently unknown. While NC has been implicated in interaction with actin [182, 183], this does not account for uropod localization of Gag-LZ in which NC was replaced with a heterologous leucine zipper [158, 172]. As Gag multimerization recruits and stabilizes lipid rafts and TEMs at assembly sites (discussed earlier), it is conceivable that reorganization of these microdomains, as well as cellular proteins associated with these microdomains, is involved in polarized localization of Gag multimers to uropods and subsequently to the VS. In support of this hypothesis, markers for both microdomains are found to accumulate at uropods [169] and VSs [110, 146, 159, 160]. Indeed, in HIV-1-expressing T cells, both a raft marker CD59 and a tetraspanin CD81 copolarize with Gag to uropods. However, using a T-cell line that polarizes spontaneously, we observed that Gag copatches with CD59 only to a very low extent prior to cell polarization (GNL unpublished data). Even within uropods, copatching between Gag and CD59 was poor (GNL unpublished data). Copatching between Gag and CD81 was shown to be higher but still at a modest level [158]. In contrast, uropod markers PSGL-1, CD43, and CD44 strongly copatch with Gag both before and after polarization [158] (GNL unpublished data). Therefore, at least in these T cells, Gag appears to associate predominantly with a specific microdomain enriched in uropod-directed proteins (uropod-directed microdomain or UDM), which is likely to be distinct from CD59-positive lipid rafts. 

One can postulate that in T cells Gag multimerization induces recruitment of UDMs more efficiently than that of lipid rafts or perhaps TEMs. UDM association may in turn promote association between Gag multimers and actin flow and thereby facilitate Gag localization to the uropod (Figure 1(b)). In support of this possibility, PSGL-1 comigrates with Gag toward the uropod as T cells polarize [158]. This possibility is further supported by the observation that actin-binding proteins such as ezrin and moesin, which are found in HIV-1 virions [90], bind cytoplasmic domains of several uropod-specific transmembrane proteins and promote localization of these proteins to uropods [169]. Alternatively, it is possible that while PSGL-1 and other UDM proteins are recruited to Gag multimers, Gag might not require UDM association for localization to the uropod. In such case, recruited UDM proteins may serve other functions in cell-to-cell transmission. Elucidating the mechanism by which Gag multimers associate with UDMs will likely allow us to determine the potential role of this association in Gag localization and cell-to-cell HIV-1 transmission.

## 5. Future Perspectives

The plasma membrane microdomains that constitute virus assembly sites have been frequently depicted as stable preformed platforms. However, a more nuanced view of plasma membrane compartmentalization is that they exist along a continuum of size and stability. On one end, large domains such as the Gag-laden uropod surface may serve as a preformed stable structure poised to form cell-cell junctions or VSs. On the other extreme, microdomains are submicroscopic, dynamic, and unstable unless protein-protein and protein-lipid interactions drive their stabilization. At least for HIV-1, it is increasingly clear that Gag multimerization and/or membrane curvature reorganizes plasma membrane microdomains at assembly sites. With this new view, a number of new questions arise: What are the characteristics specific to virus-reorganized microdomains compared to those of original individual microdomains? What is the nature of association (or lack thereof) between monomeric Gag and microdomains? Do other enveloped viruses alter microdomain organization at their assembly sites, and if so, what are the differences in composition and function among these virus-reorganized microdomains?

Cellular proteins and lipids that specifically associate with membrane microdomains of virus assembly sites affect HIV-1 particle production and infectivity, either positively (e.g., cholesterol, see [5] for a review; sphingolipids [184]) or negatively (e.g., tetraspanins; see above). Incorporation of viral proteins such as Env into virus particles may also be modulated by microdomains [1, 2]. To fully understand incorporation of these molecules into virus particles, it is crucial to elucidate the mechanisms by which Gag multimerization reorganizes microdomains. Although even Gag derivatives with heterologous membrane-binding domains can induce coalescence of lipid rafts and TEMs, membrane-binding domains of Gag may still modulate compositions of reorganized microdomains via molecular interactions. In this regard, it is interesting to note that a highly basic protein can induce formation of a microdomain enriched in acidic lipids, which in turn attract other basic proteins [185, 186]. As Gag and other viral structural proteins contain highly basic regions, it is conceivable that multimerization of these viral proteins may induce acidic lipid clustering and thereby trigger association of basic-region-containing proteins to assembly sites. Whether such indirect mechanism, in addition to direct protein-protein interactions, modulates microdomain compositions will potentially be of functional significance. 

Less well-characterized functions for reorganized microdomains include contribution to polarized localization and cell-to-cell transmission. On this front, future studies need to be directed to understanding (1) the relationships among UDMs, lipid rafts, and TEMs, (2) the mechanism by which Gag multimerization facilitates association of Gag with the retrograde actin flow, and (3) the role for UDM proteins in polarized localization and VS functions. These studies will help us further understand molecular mechanisms that facilitate VS formation and cell-to-cell transmission.
